# Factors influencing UK veterinarians’ decisions to leave equine clinical practice

**DOI:** 10.1002/vetr.5429

**Published:** 2025-06-02

**Authors:** Charlotte Rigby, Alison Prutton

**Affiliations:** ^1^ School of Veterinary Medicine University of Surrey Guildford UK

## Abstract

**Background:**

Recruitment and retention of equine veterinarians presents an ongoing challenge for employers. Recent research has highlighted factors that contribute to attrition across the profession, but further understanding of the specific challenges faced in the equine sector is needed. This study builds on existing research by exploring the relative impact of different factors on veterinarians’ decisions to leave equine clinical practice.

**Methods:**

Veterinarians who had left equine clinical practice in the UK within the previous 10 years were invited to participate in an anonymous online survey. The participants were asked about the extent to which various factors influenced their decision to leave, and about the kind of job roles they were pursuing instead.

**Results:**

Both work‐related and non‐work‐related (personal) factors had an important impact on the decision to leave equine clinical practice, and there was often interplay between the two. The three factors with the most significant impact were work related. These were ‘excessive workload/unsustainable work schedules’, ‘the requirement for out‐of‐hours work’ and ‘lack of opportunity for professional growth’. The most important non‐work‐related factors were ‘lack of personal time’ and ‘alternative professional interests’. In comparison to existing research from the entire veterinary profession, the requirement for out‐of‐hours work and a lack of opportunity for professional growth appeared to be particularly relevant for the equine sector.

**Limitations:**

The number of participants was relatively small (*n* = 33), and limited demographic data were collected; therefore, the results cannot be extrapolated to different demographic groups.

**Conclusion:**

This information may help guide decision making to improve retention of a resilient equine veterinary workforce.

## INTRODUCTION

Recruitment and retention of veterinarians in the UK is currently an issue of concern and has been the source of much discussion, debate and research in recent years.^1^, ^2^, ^3^ Challenges have been compounded by Brexit and the COVID‐19 pandemic.^4^ In a 2020 UK study, 91.4% of employers felt that it was ‘difficult’ or ‘very difficult’ to recruit an experienced veterinarian.^3^

The equine sector of the profession is no exception to these issues, and the topic of recruitment and retention has featured regularly in discussions at conferences and in the equine veterinary press and literature.^5^, ^6^, ^7^, ^8^, ^9^, ^10^, ^11^ A recent study investigating factors affecting recruitment and retention across the veterinary profession in the UK revealed that the most common reasons for practitioners to consider looking for a new position were work‒life balance, management and salary.^3^ Another study, looking at job satisfaction and retention of equine veterinarians in the USA, found that a lack of work‒life balance, long hours, lower‐than‐expected pay and issues with discrimination and bias were the main barriers to retention.^11^ It is pertinent to expand on existing research by gaining an understanding of how various factors contribute to decision making in relation to attrition of equine veterinarians in the UK, and whether this differs compared to other sectors of the veterinary profession.

In addition to the discussed challenges around retention, recent evidence suggests that UK veterinary students perceive barriers that can dissuade them from entering a career in equine practice.^12^ These perceived barriers include not having an equine background, limitations in job prospects (e.g., competitiveness of the equine sector), lack of experience or confidence with equine practice and the challenges of the role itself (such as high client expectations and poor work‒life balance). A survey by the American Association of Equine Practitioners revealed that fewer students are entering the equine profession in the USA,^13^ and it is feasible that this could also be a risk in the UK unless students can be sufficiently supported and encouraged to enter the sector. Understanding whether such perceptions and barriers hold true for veterinarians who have left the equine sector is helpful to inform the profession and identify areas for improvement.

This study aimed to explore the factors that contributed to veterinarians’ decisions to leave their equine clinical role and to establish the types of roles that they tended to pursue instead. Understanding the extent of the impact that different factors may have could help to inform the profession and aid the implementation of measures to improve retention going forward.

## METHODS

### Survey

This study used an anonymous survey (JISC Online Surveys) to explore the relative importance of factors that contributed to equine veterinarians’ decisions to leave their field of work. The survey was open from 12 February to 13 March 2024. Participants were recruited through volunteer sampling by distributing the survey, with permission, through veterinary‐related social media groups (Vets Stay Go Diversify and Equine Vet Voices), to veterinary practices partnered with the University of Surrey via a newsletter, and to teaching staff at the University. An incentive of entry into a prize draw for a voucher for an online retailer was offered to encourage participation. Informed consent was obtained. To be included in the study, participants were required to have previously worked as an equine veterinarian in the UK for a period of 6 months or more, and to have subsequently made the decision to leave that role within the last 10 years.

Demographic information on gender and the length of time participants had worked in equine practice was collected. The participants were asked to rate their level of agreement on a five‐point Likert scale (strongly disagree to strongly agree) to indicate how much each of 14 factors influenced their decision to leave equine practice. The factors were selected based on previously published work^14^ and modified slightly to reflect challenges that may be specific to the equine sector, such as the relatively high risk of injury^15^ and the increased need for out‐of‐hours working.^16^ The survey questions were divided into two distinct sections: work‐related factors (*n* = 9) and non‐work‐related (i.e., personal) factors (*n* = 5). Free‐text questions at the end of each section invited participants to state any additional work‐related or non‐work‐related factors, not already listed in the survey, which may have had an impact on their decision to leave equine practice. Finally, participants were asked to provide information about the type of role they had entered (or were planning to enter) after leaving equine practice by selecting one of the following options: ‘non‐equine clinical practice’, ‘veterinary industry (non‐clinical)’, ‘veterinary education’, ‘still deciding/unsure’ or ‘other’. Participants who selected ‘other’ were directed to a free‐text question allowing them to expand on their chosen career path.

### Data analysis

Microsoft Excel and Jamovi were used to analyse the data. Likert‐scale data were converted to a numerical scale (1 = strongly disagree, 2 = disagree, 3 = neutral, 4 = agree, 5 = strongly agree) and descriptive statistics were used to assess and compare the impact of each influencing factor on veterinarians’ decisions to leave equine clinical practice. The data were tested for normality using a Shapiro‒Wilk test and were shown to be non‐parametric; therefore, median responses were primarily used for analysis purposes. Mann‒Whitney *U*‐tests were used to analyse the relationship between gender and the extent to which each factor impacted on participants’ decisions to leave, and also the relationship between the number of years participants had worked in equine clinical practice and the extent to which each factor impacted on their decision to leave. Statistical significance was set at a *p*‐value of 0.05 or less.

Thematic analysis was used for qualitative assessment of free‐text data, in line with previously described methods.^17^, ^18^, ^19^ Prior to thematic analysis, responses from both free‐text questions (regarding additional work‐related and non‐work‐related factors that influenced their decision to leave) were compiled. Coding of the response data into themes was then independently completed by both authors. The responses were read, and once the author was familiar with the content, the comments were grouped into themes that explained the dataset. Themes could include the pre‐determined work‐related and non‐work‐related factors or novel themes, where these were identified. For example, if a participant responded ‘Did a PhD as fancied a new challenge’ to the question about additional factors that influenced their decision to leave equine practice, this was categorised into the pre‐existing factor ‘alternative professional interests’. The two authors then discussed their results to reach a consensus on the identified themes.

## RESULTS

A total of 33 participants completed the survey, 27 of whom were female (81.8%). There was a broad distribution in the number of years of experience in equine practice, with 24% of participants having worked for 0.5‒5 years, 36% for 5‒10 years, 15% for 10‒15 years and 24% for 15 years or more.

### Impact of different factors on participants’ decisions to leave equine practice

**FIGURE 1:**
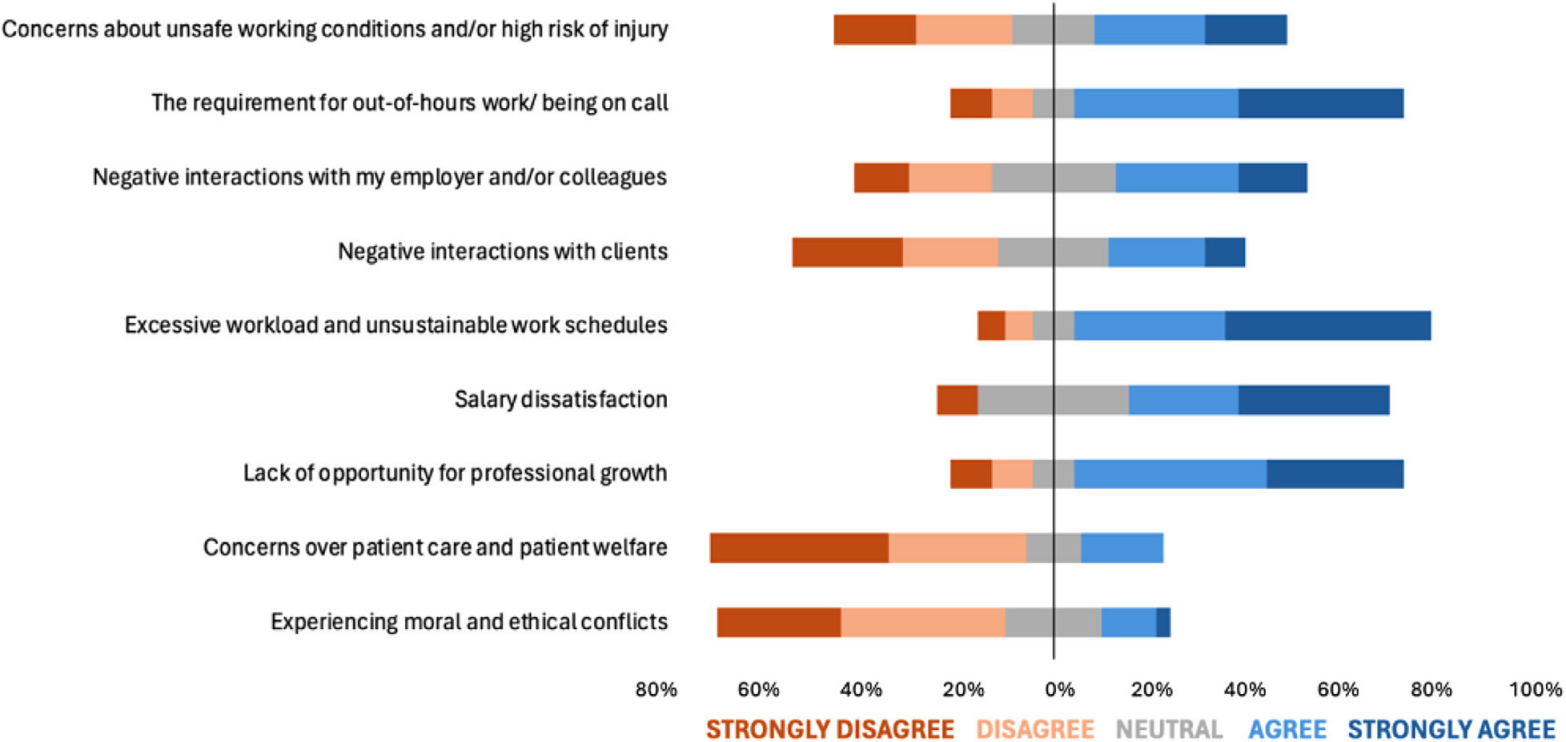
Diverging stacked bar chart illustrating survey responses (*n* = 33) to Likert‐scale questions regarding the extent to which nine work‐related factors influenced participants’ decisions to leave equine clinical practice

**FIGURE 2:**
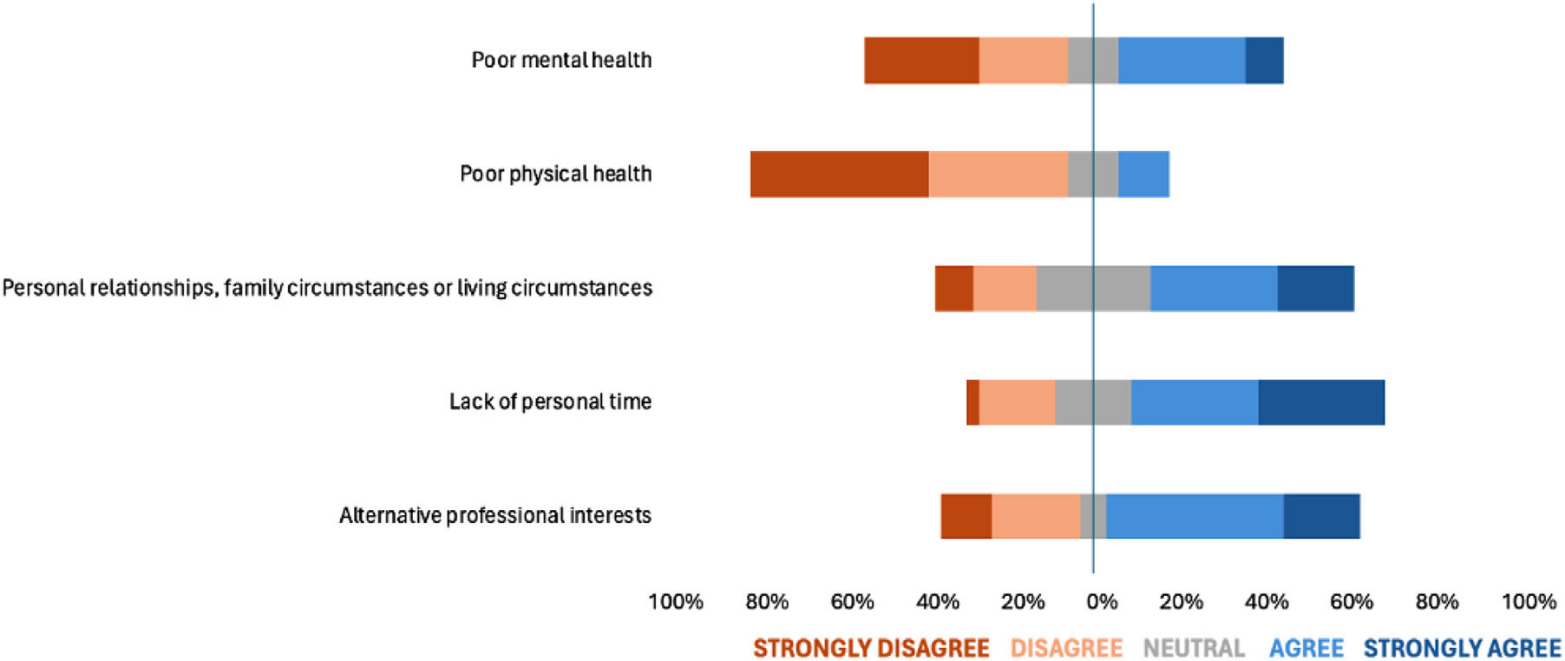
Diverging stacked bar chart illustrating survey responses (*n* = 33) to Likert‐scale questions regarding the extent to which five non‐work‐related (personal) factors influenced participants’ decisions to leave equine clinical practice

Responses to Likert‐scale questions on how work‐related factors influenced participants’ decisions to leave equine practice are shown in Figure 1. Responses to Likert‐scale questions on the impact of non‐work‐related factors are shown in Figure 2. The highest median response was ‘agree’, and six factors generated this response (four work‐related and two non‐work‐related factors). Table 1 shows the ranking of work‐related and non‐work‐related factors based on median and mean responses. When mean response was used for analysis, the top three factors that influenced veterinarians’ decisions to leave equine clinical practice were all work‐related factors. These factors were: ‘Excessive workload and unsustainable work schedules’, ‘The requirement for out‐of‐hours work/being on call’ and ‘Lack of opportunity for professional growth’. Mann‒Whitney *U*‐tests revealed that ‘experiencing moral and ethical conflicts’ was the only factor to show a statistically significant relationship with gender (*p* = 0.034), with males being more likely to strongly disagree that experiencing moral and ethical conflicts influenced their decision to leave equine practice, whereas females tended to take a more neutral stance. There was no statistically significant relationship between the number of years in equine practice and the extent to which any work‐related or non‐work‐related factors influenced participants’ decisions to leave.

**TABLE 1:** Ranked median and mean responses to Likert‐scale questions regarding the impact of nine work‐related and five non‐work‐related (personal) factors on participants’ decisions to leave equine practice

	Median	Mean
**Work‐related factors**		
Excessive workload and unsustainable work schedules The requirement for out‐of‐hours work/being on call Lack of opportunity for professional growth Salary dissatisfaction	4 (agree)	4.06 3.82 3.76 3.73
Negative interactions with my employer and/or colleagues Concerns about unsafe working conditions and/or high risk of injury Negative interactions with clients	3 (neutral)	3.15 3.03 2.70
Experiencing moral and ethical conflicts Concerns over patient care and welfare	2 (disagree)	2.72 2.09
**Non‐work‐related factors**		
Lack of personal time Alternative professional interests	4 (agree)	3.67 3.33
Personal relationships, family circumstances or living circumstances Poor mental health	3 (neutral)	3.33 2.73
Poor physical health	2 (disagree)	1.94

### Qualitative analysis of free‐text responses

There were 15 responses to the free‐text question ‘If you wish to, please state any other work‐related factors that may have had an impact on your decision to leave equine clinical practice’ and eight responses to the question ‘If you wish to, please state any non‐work‐related/personal or lifestyle factors that may have had an impact on your decision to leave equine clinical practice’. Responses from both questions (*n* = 23) were compiled for coding and thematic analysis. In many cases, responses could be categorised into a theme that aligned with one of the existing 14 work‐related and non‐work‐related factors that formed the basis of the Likert‐scale questions. In addition, several emergent themes were identified. These included a lack of employer flexibility, work‐related stress and/or anxiety, and unsupportive management.

The most commonly identified themes from free‐text analysis were as follows:

- excessive workload or unsustainable work schedules (five responses);
- personal relationships, family circumstances or living circumstances (five responses);
- lack of employer flexibility (five responses);
- the requirement for out‐of‐hours working/being on‐call (four responses);
- poor mental health (four responses);
- lack of opportunity for professional growth (three responses);
- salary dissatisfaction (three responses);
- negative interactions with employers and/or colleagues (three responses).

Out of the eight themes listed above, only ‘lack of employer flexibility’ was not included in the original list of 15 factors. Several participant responses illustrated the inherent inter‐relationship of work‐related and non‐work‐related factors, with responses such as the following:

“Work‐related stress was strongly impacting my life outside of work”. (Participant 7)

“Impact of sleep disruption during on‐call work on child and partner”. (Participant 9)

### Alternative career choices

**FIGURE 3:**
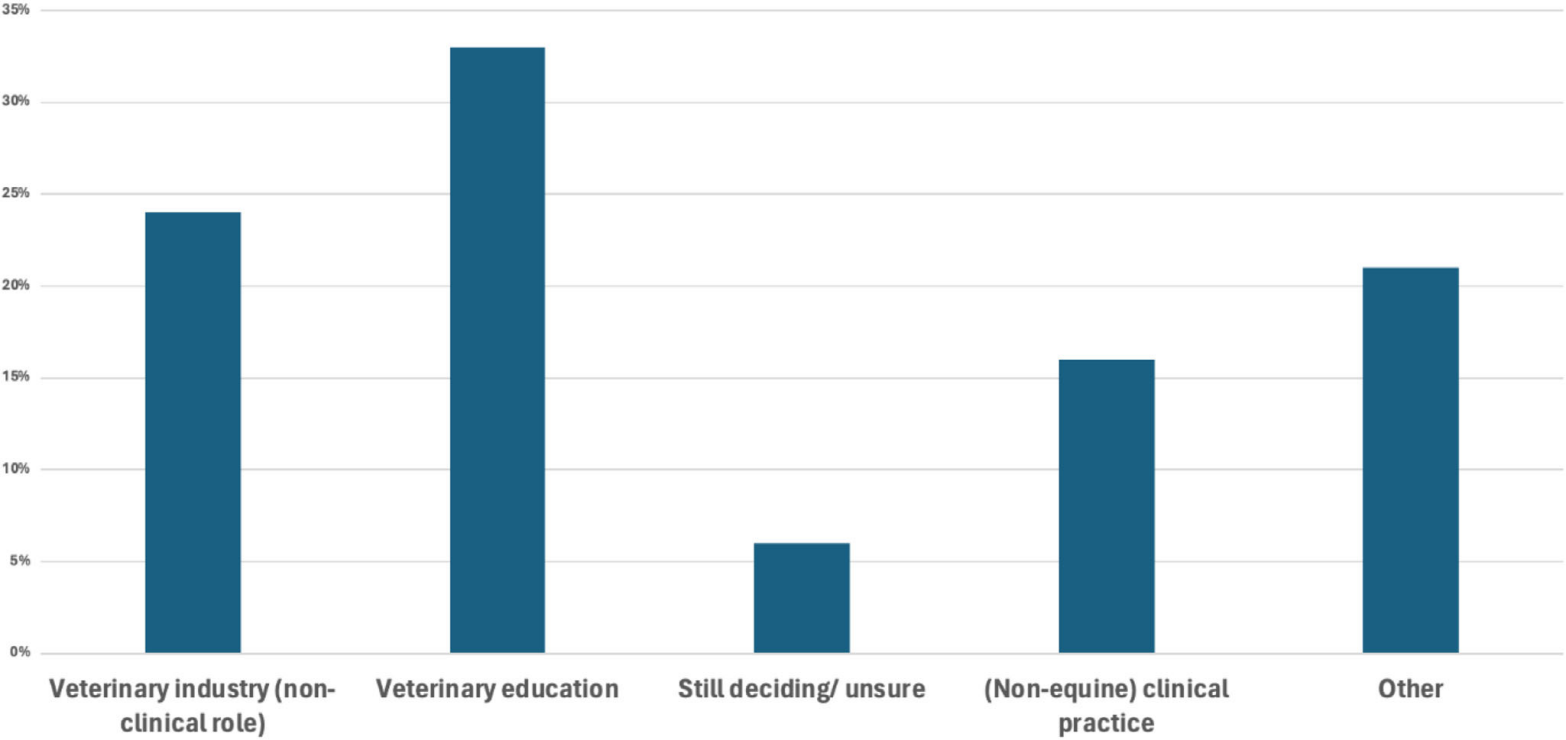
Bar chart showing participant responses to the question ‘Please indicate the type of role you entered (or are planning to enter) after leaving equine clinical practice’

All 33 participants provided an answer to the question ‘Please indicate the type of role you entered (or are planning to enter) after leaving equine clinical practice’ (Figure 3). For the seven participants who selected the category ‘other’, the free‐text responses included unspecified non‐veterinary roles (*n* = 2), human pharmaceutical industry, equine imaging, academia (PhD), medical communications and pathology.

## DISCUSSION

In keeping with previous research relating to the veterinary profession in its entirety,^3^, ^14^ this study shows that a combination of work‐related and non‐work‐related (personal) factors influenced participants' decisions to leave equine practice. Six out of 14 factors (four work‐related and two non‐work‐related) had a median response of ‘agree’ in the Likert‐scale responses, highlighting the multifactorial nature of veterinarians’ decisions to leave equine practice. Previous work on retention of veterinary and other healthcare workers^14^, ^20^, ^21^, ^22^ has indicated that both work‐related and non‐work‐related factors are important, and that they are not independent from each other, indicating a need for multifactorial approaches to interventions to maximise retention. The inherent inter‐relationship between work‐related and non‐work‐related factors has previously been established in the literature^14^ and became distinctly evident during analysis of free‐text responses in our survey, with responses such as:

“If I want to progress, I need to do a residency, and I can't afford to work for that salary and hours with dependents”. (Participant 4)

Based on mean responses to the Likert‐scale questions, the three factors that appeared to have the most impact on decisions to leave were excessive workload and unsustainable work schedules, the requirement for out‐of‐hours work/being on call and a lack of opportunity for professional growth. These findings are largely consistent with previous research relating to the UK veterinary profession^3^, ^14^, ^23^, ^24^; however, this study indicates that the additional requirement for out‐of‐hours working/being on‐call and the lack of opportunity for professional growth appear to be especially important in equine practice compared to other sectors of the veterinary profession.

The findings that excessive workload/unsustainable work schedules and the requirement for out‐of‐hours work/being on call were important factors in veterinarians’ decisions to leave equine practice are in keeping with existing evidence.^23^ A UK survey showed that equine veterinarians tend to work longer hours than small animal, farm animal and mixed animal colleagues. A total of 90.3% of equine veterinarians reported that they carry out out‐of‐hours work, compared to 48.8% of small animal colleagues.^16^ There may be opportunities for employers to address these concerns to some extent through shared out‐of‐hours provision across practices or other forms of caseload sharing.^11^ The use of dedicated equine out‐of‐hours providers has become increasingly popular in recent years.^24^ Some employers may be reluctant to change their approach to out‐of‐hours provision due to challenges around cost, lack of case continuity and impact on client satisfaction, but with the current recruitment and retention issues faced by the sector, these challenges must be balanced with improved employee satisfaction associated with a better work‒life balance. Salary dissatisfaction was also a seemingly important factor in participants’ decisions to leave equine practice (median ‘agree’). This is consistent with previous work both in the profession as a whole^14^, ^23^ and within the equine sector in other countries.^11^ Interventions such as higher pay and a work culture shift towards caseload sharing^11^ may help to balance emergency care provision and employee satisfaction.

The findings regarding the impact of a lack of opportunity for professional growth on participants’ decision to leave equine practice reflect a need for employers and managers to ensure the availability and clear communication of adequate and varied job progression opportunities. The veterinary profession, including the equine sector, has undergone rapid corporatisation in the past decade, and it is possible that the associated shift away from the more traditional progression pathway towards practice partnership may have affected equine veterinarians’ perceptions of progression opportunities.^25^ On the other hand, corporate ownership can also enhance career progression through the provision of graduate training schemes, continuing education and senior management opportunities.^25^ Regardless of the type of practice, whether corporate owned or independent, professional growth opportunities remain an important area for employers to consider when it comes to maximising the retention of equine veterinarians.

The most commonly reported length of time participants in this study had worked in equine practice was 5‒10 years. This is in line with the findings of the BVA ‘Voice of the Veterinary Profession’ survey, which revealed that the most common time to leave practice is 7 years after qualification.^5^, ^26^ Research from both the veterinary^3^ and human healthcare^21^ fields indicates that having more working years (i.e., more experience) overall tends to increase the chances of employee retention. The authors of the present study theorise that participants from the 5‒10 years category may have had a sufficiently long time to gain some experience in their field and to better understand their career interests and aspirations while also having experienced some of the limitations and challenges of the role. Family commitments have previously been cited to be one of the main reasons that veterinarians would consider leaving practice,^3^ and it follows that this 5‒10 year timing for leaving the profession may coincide with increasing family commitments and thus increased challenges in maintaining an effective work‒life balance.

The majority of participants in this study were female, which is consistent with the UK equine veterinary profession, which was approximately 73.6% female in 2020.^27^ It has been shown in some studies that female veterinarians were more likely to leave clinical work than males,^3^ whereas others have shown the opposite.^23^ However, being female has been shown to be a positive predictor for work exhaustion, interpersonal disengagement and burnout.^28^ Further research is required to fully understand the contribution of gender to the decision to leave equine clinical practice, but undoubtedly, employer interventions should aim to ensure equal opportunities for professional growth and sustainable work schedules that allow employees to balance work and family commitments to maximise retention.

The factor ‘Negative interactions with my employer and/or colleagues’ did not appear to be a particularly strong factor in participants’ decision to leave equine practice, based on the Likert‐scale responses (median ‘neutral’). Employer and colleague interactions were combined into one potential factor in this study, but future work should explore these as separate, independent entities to better understand how employer/management interactions affect retention. There were no Likert‐scale questions that specifically asked participants about the impact of management approaches on their decision to leave; however, free‐text responses indicated that management was perceived to be an important issue. Themes such as unsupportive or poor management, challenges around pregnancy and maternity leave management and discrimination, negative perceptions of part‐time workers (by managers and colleagues), lack of sick pay and unjustifiable pricing structures emerged. Lack of employer flexibility was the third most common theme identified in thematic analysis of free‐text responses, which ties in with previous work that cites a lack of work‒life balance as an important factor in the decision to remain in or leave clinical practice.^3^, ^23^ Lack of employer flexibility also relates to the current study's results around the impact of excessive workload and unsustainable work schedules, and the requirement for out‐of‐hours work/being on call. Future research should focus on trying to further understand how management issues relate specifically to equine practice and what interventions should be employed to improve perceptions and management approaches.

The responses to the Likert‐scale questions indicated that poor mental health was not a major influencing factor in participants’ decision to leave equine clinical practice (median ‘neutral’). Although this is reassuring for both current and future equine veterinarians, it was somewhat contradicted by the free‐text responses, where mental health concerns or work‐related stress and anxiety were mentioned by four participants, two of whom described poor management of these issues. It is documented that veterinarians may describe higher levels of depression, anxiety, stress and burnout compared to the general population, and that this varies according to gender, background, type of practice and years after graduation.^20^ The impact of mental health on retention of equine veterinarians, and management strategies to help employees suffering from mental health issues, deserves further investigation.

Concerns about unsafe working conditions and/or high risk of injury (median ‘neutral’) and poor physical health (median ‘disagree’) did not appear to be factors of particularly high importance in participants’ decisions to leave equine practice, despite the reported high injury rate of the profession.^15^ This is an encouraging finding, and there have been positive steps in recent years within the profession towards mitigating the inherent risks associated with the role of equine veterinarians to protect employees’ and clients’ physical wellbeing.^29^, ^30^ However, results around perceptions of safety and risk of injury should be interpreted with caution. A recent study revealed that equine and production animal veterinarians have a high threshold before acknowledging that an incident is a work‐related injury, with some harms considered as ‘everyday norms’, leading to potential barriers to reporting.^31^

Experiencing moral and ethical conflicts and concerns over patient care and welfare (both median ‘disagree’) did not appear to have a strong influence on participants’ decision to leave equine clinical practice. Veterinarians can experience moral or ethical conflict, which can lead to moral distress.^28^, ^32^, ^33^ The concept of moral distress has been widely studied in human healthcare professionals,^34^, ^35^ but there is limited evidence to understand the impact of moral distress on attrition of veterinarians.^32^ Some literature suggests that there is a link between moral distress and attrition of veterinarians,^14^, ^28^, ^36^ but the results of our study do not support moral distress as a significant factor in veterinarians’ decisions to leave equine clinical practice. Further research should aim to establish and quantify the effects of moral distress on job satisfaction and attrition in different sectors of veterinary medicine and assess its impact on equine clinical practice in particular.

Participant gender had a statistically significant relationship with the way participants responded to the Likert‐scale question on ‘experiencing moral and ethical conflicts’, with males being more likely to strongly disagree that this factor influenced their decision to leave equine practice, and females tending towards a more neutral response. This apparent gender difference in the experience of moral distress is consistent with literature from the nursing profession^37^; however, it should be interpreted with caution in this study, as there was a very small number of male participants, which may reduce the statistical power of the finding.

Negative interactions with clients did not appear to be a factor of high importance in the decision to leave equine clinical practice (median ‘neutral’). This finding is contradictory to the reportedly commonly held perception by UK veterinary students of ‘high expectations’ and an ‘unwelcoming’ or ‘elitist’ manner of equine clients, which are seen as barriers to entering a career in equine practice.^12^ The results from this study and other research into client interactions in equine practice could aid veterinary educators and the equine industry to support and increase the equine veterinary workforce by working towards changing these negative perceptions to make the profession more appealing to students. There is a need to attract students from non‐equine backgrounds, and to improve diversity, equity, inclusion and belonging within the profession.^11^

After leaving their role in equine clinical practice, the majority of participants entered non‐clinical roles within the veterinary industry, veterinary education roles or various ‘other’ veterinary‐related roles. However, there were also several participants who entered non‐equine clinical roles or pursued careers outside of the veterinary industry. The diversity of roles pursued demonstrates the versatility and transferability of veterinarians' skills.

A large proportion of the research related to veterinary attrition considers the profession as a whole, whereas this study was specific to equine clinical practice. Furthermore, previous literature has mainly focused on job satisfaction and reasons why veterinarians currently working in practice might consider leaving their roles. This study is one of very few involving participants who have already left equine practice, and who are therefore able to reflect on their time in practice with hindsight and lived experience. Exit interviews can be a useful tool,^38^, ^39^ which may be underutilised in the veterinary industry. While beneficial in terms of their unique insight, the use of veterinarians who had already left the equine profession presented some challenges in terms of recruitment, resulting in a relatively small sample size, which is a limitation of the study.

Data on demographic information beyond gender and years of experience in equine practice were not collected in this study; therefore, the results cannot be extrapolated to individual demographic groups. There is potential for future studies establishing how demographics influence attrition of equine veterinarians to build on recent work^11^ indicating that issues with discrimination and bias can act as barriers to equine veterinary retention.

Another limitation of the study is that volunteer sampling was used. This approach can pose a risk of self‐selection bias^40^; for example, participants may be more likely to respond if they have feelings of dissatisfaction. Likert‐scale responses can also be subject to distortion from a variety of causes. Central tendency bias may occur, whereby respondents avoid the use of extreme response categories, and respondents may agree with statements simply because the statement has been presented.^41^ Questions on the specific impact of management style/approach were not included in the Likert‐scale questions, but concerns around management were commonly cited in the free‐text portion of the survey, indicating that management issues may have been more impactful than it appeared based on Likert‐scale results and should therefore be considered further in future studies. Distribution of the survey to academic staff may have resulted in an over‐representation of participants who moved into veterinary education roles. In addition, distribution to veterinary‐related social media sites may have resulted in a skewed proportion of how many participants entered non‐equine or non‐clinical roles but stayed within the veterinary industry. Despite these limitations, the findings were compatible with previous literature and provide further insight into the specific challenges faced within the equine sector.

## CONCLUSION

The outcomes of this study provide a useful insight for equine veterinary employers into the most important factors that they can focus on to help improve retention. The study also provides information that may increase awareness among equine veterinarians and help prepare veterinary students thinking of entering the profession. Future research should use larger sample sizes to explore the impact of demographic data such as background, family commitments and gender and further characterise how management practices affect retention. Stakeholder focus groups could help to identify solutions and interventions that can be implemented to enable a sustainable and fulfilled equine veterinary workforce.

## AUTHOR CONTRIBUTIONS

The authors were jointly responsible for data collection, analysis and write‐up.

## CONFLICT OF INTEREST STATEMENT

Neither of the authors has any financial or personal relationships that could inappropriately influence or bias the content of the paper.

## FUNDING INFORMATION

The authors received no specific funding for this study.

## ETHICS STATEMENT

The study was approved by the Ethics Committee and the Research Integrity and Governance Office at the University of Surrey (SAGE‐HDR 1046015‐1045997‐116864347).

## Data Availability

The data will be made available upon reasonable request.
